# PaP1, a Broad-Spectrum Lysin-Derived Cationic Peptide to Treat Polymicrobial Skin Infections

**DOI:** 10.3389/fmicb.2022.817228

**Published:** 2022-03-10

**Authors:** Ryan D. Heselpoth, Chad W. Euler, Vincent A. Fischetti

**Affiliations:** ^1^Laboratory of Bacterial Pathogenesis and Immunology, The Rockefeller University, New York, NY, United States; ^2^Department of Medical Laboratory Sciences, Hunter College, New York, NY, United States; ^3^Department of Microbiology and Immunology, Weill Cornell Medical College, New York, NY, United States

**Keywords:** lysin, antimicrobial peptide, antibiotic resistance, bacteriophage, broad-spectrum, *Pseudomonas aeruginosa*, *Staphylococcus aureus*, polymicrobial

## Abstract

Most skin infections, including those complicating burns, are polymicrobial involving multiple causative bacteria. Add to this the fact that many of these organisms may be antibiotic-resistant, and a simple skin lesion or burn could soon become life-threatening. Membrane-acting cationic peptides from Gram-negative bacteriophage lysins can potentially aid in addressing the urgent need for alternative therapeutics. Such peptides natively constitute an amphipathic region within the structural composition of these lysins and function to permit outer membrane permeabilization in Gram-negative bacteria when added externally. This consequently allows the lysin to access and degrade the peptidoglycan substrate, resulting in rapid hypotonic lysis and bacterial death. When separated from the lysin, some of these cationic peptides kill sensitive bacteria more effectively than the native molecule *via* both outer and cytoplasmic membrane disruption. In this study, we evaluated the antibacterial properties of a modified cationic peptide from the broad-acting lysin PlyPa01. The peptide, termed PaP1, exhibited potent *in vitro* bactericidal activity toward numerous high priority Gram-positive and Gram-negative pathogens, including all the antibiotic-resistant ESKAPE pathogens. Both planktonic and biofilm-state bacteria were sensitive to the peptide, and results from time-kill assays revealed PaP1 kills bacteria on contact. The peptide was bactericidal over a wide temperature and pH range and could withstand autoclaving without loss of activity. However, high salt concentrations and complex matrices were found to be largely inhibitory, limiting its use to topical applications. Importantly, unlike other membrane-acting antimicrobials, PaP1 lacked cytotoxicity toward human cells. Results from a murine burn wound infection model using methicillin-resistant *Staphylococcus aureus* or multidrug-resistant *Pseudomonas aeruginosa* validated the *in vivo* antibacterial efficacy of PaP1. In these studies, the peptide enhanced the potency of topical antibiotics used clinically for treating chronic wound infections. Despite the necessity for additional preclinical drug development, the collective data from our study support PaP1 as a potential broad-spectrum monotherapy or adjunctive therapy for the topical treatment of polymicrobial infections and provide a foundation for engineering future lysin-derived peptides with improved antibacterial properties.

## Introduction

Chronic wound infections are generally polymicrobial in nature (Bowler et al., 2001; Kirketerp-Moller et al., 2008). The interactions between the different microorganisms in these diverse communities have significant ramifications on disease progression and clinical outcome, with higher mortality rates observed than monomicrobial infections due to increased pathogenicity, biofilm formation and antibiotic resistance (Short et al., 2014; Nabb et al., 2019). The increased prevalence of antibiotic resistance in these microenvironments has been selected for by drug overuse and misuse, which is a reality that poses a serious threat to global public health (Ventola, 2015). In the United States, there are more than 2.8 million antibiotic-resistant infections annually, resulting in over 35,000 deaths (Centers for Disease Control and Prevention, 2019). Pharmaceutical research and development have failed to meet the clinical need for novel antibiotics because of challenges encompassing clinical development, as well as scientific, regulatory and economic issues (Tacconelli et al., 2018). The absence of a coordinated discovery platform over the past 50 years has afforded bacteria the opportunity to continually develop resistance to existing antibiotics. By using a combination of resistance mechanisms, the rapid evolution and dissemination of multidrug-resistant (MDR), extensively drug-resistant (XDR), and pandrug-resistant (PDR) bacteria further raises the threat level (Magiorakos et al., 2012). Importantly, in addition to saving lives, antibiotics enable modern medicine. The absence of effective antibiotics would have serious implications on medical procedures, such as surgery, dialysis, chemotherapy and organ transplantation (Lewis, 2020).

Membrane-acting compounds represent a class of bacterial therapeutics that are advantageous due to a lack of resistance formation and their ability to exhibit potent antibacterial activity against dormant bacteria (Lewis, 2020). Two examples of approved membrane-acting drugs are daptomycin and the polymyxins. For Gram-positive (GP) bacterial infections, daptomycin is a calcium-dependent cyclic lipopeptide that inserts into the cytoplasmic membrane (CM), causing membrane disruption without entering the cytoplasm or causing bacterial lysis (LaPlante and Rybak, 2004; Hancock, 2005; Gonzalez-Ruiz et al., 2016). For Gram-negative (GN) bacterial infections, the polymyxins are membrane-acting antibiotics used clinically as drugs of last resort. Polymyxins bind phosphate groups of lipopolysaccharides (LPS) and phospholipids to promote bacterial membrane disruption, intracellular content leakage, and bacterial death (Evans et al., 1999; Li et al., 2001). Likewise, antimicrobial peptides (AMPs) of the innate immune system damage bacterial membranes using a similar mechanism of action. Their cationic properties increase the permeability of the bacterial membrane through selective electrostatic interactions with the negatively charged microbial cell membrane. The amphipathic nature of AMPs destabilizes the membrane *via* pore formation by inserting their hydrophobic segment into the lipid bilayer, resulting in cell lysis and death (Zanetti, 2004; Som et al., 2008; Guo et al., 2018; Klubthawee et al., 2020). Despite their antibacterial potency, an unfortunate characteristic of many membrane-acting antimicrobials is their considerable cytotoxic effects on eukaryotic cells, limiting their therapeutic dosing range (Falagas and Kasiakou, 2005; Lim et al., 2010; Boohaker et al., 2012; Justo and Bosso, 2015).

Cationic peptides from GN bacteriophage (phage) lysins represent a novel class of a membrane-acting antimicrobials. In general, lysins are peptidoglycan hydrolases used to lyse host cells in order to liberate progeny virions at the culmination of the phage infection cycle (Fischetti, 2008). Exogenously applied recombinant lysins efficiently lyse GP bacteria, an observation that highlights their potential as antimicrobials (Heselpoth et al., 2018; Danis-Wlodarczyk et al., 2021; Ho et al., 2021; Schmelcher and Loessner 2021). However, the outer membrane (OM) in GN bacteria largely prevents recombinant lysins from accessing the peptidoglycan, although there is increasing evidence that many GN lysins contain an amphipathic membrane-acting segment that permeabilizes the membrane (Lai et al., 2011; Larpin et al., 2018; Ghose and Euler, 2020). Although still a hypothesis, the cationic properties associated with this region of the lysin could potentially electrostatically interact with anionic phosphate groups located within the inner lipid A core of LPS to competitively displace stabilizing divalent cations (in particular, Mg^2+^ and Ca^2+^), which are essential for outer leaflet structural integrity. These events would permit the lysin to diffuse through the OM and cleave critical covalent bonds in the peptidoglycan structure to consequently kill the bacterial cell through hypotonic lysis.

When separated from the whole lysin molecule, the membrane-acting segment can be exploited as a cationic peptide with bactericidal properties due to its ability to disrupt the bacterial membrane. In a proof-of-concept study, P307, a 31 amino acid (aa) cationic peptide originating from the C-terminal end of the *Acinetobacter baumannii* lysin PlyF307 (Lood et al., 2015), efficiently killed *A. baumannii* (Thandar et al., 2016). The bactericidal potency of the peptide was significantly improved when an eight aa sequence (SQSRESQC) originating from the hepatitis B virus core protein was added to the C-terminus. The presence of these eight amino acids was found to enhance the activity of an arginine-rich domain of the hepatitis B virus core protein against a variety of bacterial pathogens (Chen et al., 2013). Experimental highlights associated with the modified peptide, P307_SQ-8C_, include synergism with polymyxin B, *in vitro* bactericidal activity toward biofilm-state bacteria, absence of cytotoxicity toward human cells, and log_10_-fold killing of *A. baumannii* using a mouse skin infection model (Thandar et al., 2016).

Considering P307_SQ-8C_ exhibits robust activity primarily toward *A. baumannii* only, the goal of our study was to engineer a novel lysin-derived cationic peptide with broadened bactericidal activity that covers numerous clinically relevant bacterial pathogens, with an emphasis on those predominantly found in wound infections (e.g., *A. baumannii*, *Escherichia coli*, *Proteus mirabilis*, *Pseudomonas aeruginosa* and *Staphylococcus aureus*; Bessa et al., 2015). Previously, it was shown that several *Pseudomonas* lysins were capable of comprehensively killing high priority GN pathogens (Raz et al., 2019); this finding is likely attributable to a broad membrane-acting region at the C-terminal end of each lysin. With this in mind, we evaluated the antibacterial activity of four modified peptide candidates (in parenthesis) originating from the broad-acting *P. aeruginosa* lysins PlyPa01 (PaP1), PlyPa91 (PaP91), PlyPa96 (PaP96) and PlyPa103 (PaP103). The bactericidal, biochemical and cytotoxic properties of the most potent peptide candidate, PaP1, were measured *in vitro*. The prophylactic and therapeutic antibacterial efficacy of the peptide was then assessed *in vivo* against antibiotic-resistant bacterial pathogens using a murine burn wound infection model of disease.

## Materials and Methods

### Bacterial Strains and Culture Conditions

Bacterial strains used in this study are outlined in Table 1 and were stored at −80°C. Unless stated otherwise, bacterial strains were routinely grown aerobically to logarithmic phase in either Brain Heart Infusion (GP bacteria; BD Biosciences) or Luria–Bertani (LB) medium (GN bacteria; Fisher Scientific). One exception was *Clostridium difficile*, which were grown anaerobically to logarithmic phase in reduced Brain Heart Infusion medium supplemented with 0.5% (wt/vol) yeast extract and 0.1% (wt/vol) L-cysteine.

**Table 1:** Bacterial strains used in this study.

Species	Strain	Source	Notes
*A. baumannii*	ATCC 17978	ATCC	
*A. baumannii*	ATCC BAA-1792	ATCC	MDR, CRAB
*A. baumannii*	1792–1793	HSS	MDR
*A. baumannii*	NR-17783	BEI resources, NIAID	MDR
*A. baumannii*	NR-19298-19299	BEI resources, NIAID	MDR
*B. anthracis*	ΔSterne	RUBC	pXO1^−^ pXO2^−^
*C. freundii*	ATCC 8090	ATCC	
*C. difficile*	ATCC 43255	ATCC	Toxinotype 0
*E. aerogenes*	NR-48555	BEI resources, NIAID	MDR, CRE
*E. cloacae*	NR-50391	BEI resources, NIAID	
*E. cloacae*	NR-48558	BEI resources, NIAID	CRE
*E. faecalis*	V12	RUBC	VSE
*E. faecium*	RH1	NYP/WCMC	VSE
*E. faecium*	EFSK2	ATSC	VRE
*E. faecium*	EFSK16	ATSC	VRE
*E. faecium*	EFSK33	ATSC	VRE
*E. coli*	AR531	NYU	
*E. coli*	L22-1	Satlin et al., 2018	ESBL-producing
*E. coli*	L159-1	Satlin et al., 2018	ESBL-producing
*E. coli*	B481-1	Satlin et al., 2018	ESBL-producing
*K. pneumoniae*	NR-41923	BEI resources, NIAID	
*K. pneumoniae*	ATCC 700603	ATCC	MDR, ESBL-producing
*K. pneumoniae*	NR-15410-15411	BEI resources, NIAID	*bla*_KPC_, CRE
*L. monocytogenes*	HER1184	RUBC	
*P. mirabilis*	AR397	RUBC	
*Pseudomonas* sp.	HPB0071	BEI resources, NIAID	
*P. aeruginosa*	PAO1	ATCC	
*P. aeruginosa*	443–453	NYP/WCMC	
*P. aeruginosa*	AR463-AR474	NYU	
*P. aeruginosa*	NR-51517-51530	BEI resources, NIAID	MDR
*P. entomophila*	AR455	RUBC	
*P. luteola*	AR479	RUBC	
*P. mendocina*	AR482	RUBC	
*P. oryzihabitans*	AR456	RUBC	
*P. putida*	AR478	RUBC	
*Salmonella* sp.	AR396	RUBC	Group D
*S. marcescens*	AR401	RUBC	
*S. flexneri*	ATCC 12022	ATCC	
*S. sonnei*	ATCC 25931	ATCC	
*S. aureus*	Newman	RUBC	MSSA
*S. aureus*	NRS382-384	NARSA collection	MRSA
*S. agalactiae*	090R	RULC	
*S. pneumoniae*	DCC1490	RULC	
*S. pyogenes*	D471	RULC	

*ATCC, American Type Culture Collection; ATSC, The Alexander Tomasz Strain Collection; bla_KPC_, *K. pneumoniae* carbapenemase gene; CRAB, carbapenem-resistant *A. baumannii*; CRE, carbapenem-resistant Enterobacteriaceae; ESBL, Extended-spectrum β-lactamase; HSS, Hospital for Special Surgery in New York; MDR, multidrug-resistant; MRSA, methicillin-resistant *S. aureus*; MSSA, methicillin-sensitive *S. aureus*; NARSA, Network on Antimicrobial Resistance in *S. aureus*; NIAID, National Institute of Allergies and Infectious Diseases; NYP/WCMC, NewYork Presbyterian/Weill Cornell Medical Center; NYU, New York University Langone Medical Center; RUBC, The Rockefeller University Bacterial Collection; RULC, The Rockefeller University Lancefield Collection; VRE, vancomycin-resistant Enterococcus; and VSE, vancomycin-sensitive Enterococcus*

### Peptide Synthesis and Purification

PaP1 derivate peptides were produced by Biomatik Corporation. Additional PaP1, as well as PaP91, PaP96 and PaP103, were synthesized by The Rockefeller University Proteomics Resource Center as previously described (Thandar et al., 2016). Peptides were purified to at least 90% purity. Each lyophilized peptide was resuspended in diH_2_O prior to experimental use and then stored at −80°C until needed.

### Circular Dichroism

Far-UV circular dichroism experiments were performed using a Chirascan V-100 Circular Dichroism Spectrometer (Applied Photophysics). To determine secondary structure composition, each peptide was evaluated at 0.1 mg/ml in 20 mM sodium phosphate, pH 7.0. Using a 1 mm path length quartz cuvette, far-UV spectra were recorded from 190 to 260 nm at 20°C using 1 nm steps with 5 s signal averaging per data point. Spectra were collected in triplicate and then averaged, baseline subtracted, smoothened, and finally converted to mean residue ellipticity by the Pro-Data software (Applied Photophysics).

### One Hour Killing Assays

Using a 96-well untreated microtiter plate, log-phase bacteria at approximately 10^5^–10^6^ colony-forming units per milliliter (CFU/ml) were incubated statically in 20 mM Tris–HCl, pH 7.2, with or without (untreated controls) peptides at a final concentration of 10–50 μg/ml for 1 h at 37°C. For assays evaluating peptide activity as a function of bacterial growth phase, *P. aeruginosa* strain PAO1 were initially grown at 37°C with aeration to either mid-log (OD_600nm_ of 0.5) or stationary phase (24 h growth) and then treated with PaP1 at 10 μg/ml (2.3 μM). For the dose–response killing assays using 10^6^ CFU/ml bacteria, *P. aeruginosa* strain PAO1 were incubated with peptide concentrations from 0.125 (29.0 nM) to 128 μg/ml (29.7 μM). For the subsequent dose–response killing assays using varying concentrations of bacteria, *P. aeruginosa* strain PAO1 at 10^6^–10^8^ CFU/ml were treated with PaP1 at final concentrations ranging from 10 to 1,000 μg/ml (232 μM). The temperature-based experiments were conducted by adding PaP1 at 10 μg/ml to *P. aeruginosa* strain PAO1 in buffer equilibrated at either 4°C, 25°C or 37°C. For the pH studies, the peptide at 25 μg/ml (5.8 μM) was added to *P. aeruginosa* strain PAO1 in either 25 mM acetate (pH 5), 2-morpholinoethanesulfonic acid (MES, pH 6), Tris–HCl (pH 7 and 8), N-cyclohexyl-2-aminoethanesulfonic acid (CHES, pH 9) or N-cyclohexyl-3-aminopropanesulfonic acid buffer (CAPS, pH 10). All buffering chemicals were obtained from Fisher Scientific. For the salt experiments, PaP1 at 25 μg/ml was incubated with *P. aeruginosa* strain PAO1 in 20 mM Tris–HCl, pH 7.2, supplemented with 0–500 mM NaCl (Fisher Scientific). Experiments in serum and lung surfactant involved incubating PaP1 at 25 μg/ml with *P. aeruginosa* strain PAO1 resuspended in either 0%–100% (vol/vol) inactivated human serum (HuS, pooled human male AB plasma; Sigma-Aldrich) or 0%–50% (vol/vol) beractant (Survanta; Abbvie), with buffer being used as the diluent. For the polymicrobial experiment, the different bacterial species were treated with PaP1 at 50 μg/ml (11.6 μM) and then plated on selective agar. LB agar supplemented with 32 μg/ml ceftriaxone (Sigma-Aldrich) and 4 μg/ml levofloxacin (Alfa Aesar) was used for isolating *A. baumannii* strain NR-17783, *Pseudomonas* Isolation Agar (Sigma-Aldrich) selected for *P. aeruginosa* strain NR-51517, and Mannitol Salt Agar (BD Biosciences) with 4 μg/ml oxacillin (Sigma-Aldrich) was used for isolating *S. aureus* strain NRS384. Each experiment comprised an untreated control. At the culmination of each killing assay, 100 μl and 10-fold serial dilutions from each sample were plated. Since the minimum number of countable colonies is 1 CFU in the 100 μl plated, the limit of detection for each experiment was 10 CFU/ml. Error bars correspond to ± the standard error of the mean (SEM) of triplicate experiments.

### Antibiofilm Activity

The bactericidal activity of PaP1 toward biofilm-state bacteria was measured using a previously described method (Raz et al., 2019). Briefly, an overnight culture of *P. aeruginosa* strain PAO1 was diluted 1:1,000 in tryptic soy broth (BD Biosciences) with 0.2% (wt/vol) glucose. The diluted bacteria were added to an MBEC Assay® Biofilm Inoculator 96-well plate (Innovotech) at 100 μl per well. Sterility controls were included, which consisted of growth medium absent bacteria. Biofilms were formed on the plastic pegs attached to the MBEC plate lid by incubating the plate for 24 h at 37°C with shaking at 65 revolutions per minute (RPM). Biofilms were washed twice with 20 mM Tris–HCl, pH 7.2, and then treated in buffer with or without (growth control) PaP1 at final concentrations from 1 to 256 μg/ml for 2 h at 37°C with shaking. Biofilms were again washed twice with buffer. In order to recover the biofilm-state bacteria, the MBEC plate lid was transferred to a new 96-well plate containing 200 μl per well of buffer. The plate was sonicated in a water bath for 30 min, and then 100 μl and 10-fold serial dilutions from each sample were plated. The limit of detection was 10 CFU/ml. Error bars correspond to ± SEM of triplicate experiments.

### Time-Kill Assay

Using a 96-well untreated microtiter plate, mid-log *P. aeruginosa* strain PAO1 at 10^6^ CFU/ml were incubated with or without (untreated controls) PaP1 at 10 μg/ml in 20 mM Tris–HCl, pH 7.2, for 30 min at 37°C. In 5 min intervals, an aliquot was removed from each sample. Both 100 μl and 10-fold serial dilutions from each sample were plated. The limit of detection was 10 CFU/ml. Error bars correspond to ± SEM of triplicate experiments.

### Measuring Thermal Stability

Using an Echotherm heating/chilling plate (Torrey Pines Scientific), PaP1 was incubated at 100 μg/ml in phosphate buffered saline (PBS), pH 7.4, in a microcentrifuge tube for 30 min at temperatures ranging from 4°C (unheated control) to 90°C. Incubation at higher temperatures was achieved by either boiling (100°C) or autoclaving (123°C) the peptide for 30 min. Following heat treatment, the peptide samples were immediately placed on ice for at least 10 min. To quantitate residual bactericidal activity, the peptide at a final concentration of 10 μg/ml was incubated statically with mid-log *P. aeruginosa* strain PAO1 at ~10^6^ CFU/ml in 20 mM Tris–HCl, pH 7.2, for 1 h at 37°C. An untreated control sample set was included consisting of bacteria incubated in buffer only. Finally, 100 μl and 10-fold serial dilutions from each sample were plated. The limit of detection was 10 CFU/ml. Error bars correspond to ± SEM of triplicate experiments.

### NPN Uptake Assay

Bacterial OM permeabilization by PaP1 was assessed using a N-phenyl-1-naphthylamine (NPN; Fisher Scientific) uptake assay as previously described, with modifications (Gerits et al., 2016). Briefly, mid-log *P. aeruginosa* strain PAO1 were resuspended in 20 mM Tris–HCl, pH 7.2, to an OD_600nm_ of 0.5. Using a 96-well untreated black microtiter plate (clear bottom), 100 μl bacteria were mixed with 20 μl of NPN solution (25 μM NPN final concentration) and 80 μl buffer with or without (untreated controls) PaP1 at final concentrations from 1 to 64 μg/ml (14.9 μM). The positive controls for OM permeabilization consisted of bacteria treated with 2 μg/ml polymyxin B (EMD Millipore). After 1 h at 37°C, a SpectraMax M5 microplate reader (Molecular Devices) was used to measure the increase in NPN fluorescence for each sample (excitation wavelength at 350 nm and emission at 420 nm). Data were normalized to the untreated controls. Error bars correspond to ± SEM of triplicate experiments.

### SYTOX Green Uptake Assay

The structural integrity of the bacterial CM was analyzed following peptide treatment using the SYTOX Green uptake assay. This protocol was adapted from previous studies (Chen et al., 2013; Gerits et al., 2016). Briefly, mid-log *P. aeruginosa* strain PAO1 and *S. aureus* strain Newman were resuspended in 20 mM Tris–HCl, pH 7.2, to an OD_600nm_ of either 0.5 (*P. aeruginosa*) or 0.3 (*S. aureus*). Using a 96-well untreated black microtiter plate (clear bottom), 90 μl bacteria were incubated with 10 μl SYTOX Green dye (5 μM final concentration; Invitrogen) for 30 min at room temperature in the dark. Next, an equal volume was added consisting of buffer with or without (untreated controls) PaP1 at a final concentration up to 128 μg/ml. Positive controls for CM disruption consisted of bacteria treated with 10 μg/ml melittin (EMD Millipore). After 1 h at 37°C, the microplate reader was used to measure the increase in SYTOX Green fluorescence for each sample (excitation wavelength at 485 nm and emission at 520 nm). Data were normalized to the untreated controls. Error bars correspond to ± SEM of triplicate experiments.

### Cytotoxicity Assays

The hemolytic properties of PaP1 were measured as previously described, with modifications (Heselpoth et al., 2019). Briefly, human blood originating from healthy adult donors was collected in an EDTA-containing conical tube at The Rockefeller University Hospital. This study was approved by our Institutional Review Board, and all adult subjects provided written informed consent. Human red blood cells (hRBCs) were harvested by low speed centrifugation, washed three times, and resuspended in PBS, pH 7.4, to a 10% (vol/vol) concentration. Using a 96-well untreated microtiter plate, the hRBC solution was diluted 1:1 with PaP1 at final concentrations from 0.5 to 256 μg/ml. hRBCs incubated in PBS with or without 0.1% (vol/vol) Triton X-100 represented positive and negative controls for hemolysis, respectively. Following a 4-h incubation at 37°C with 5% CO_2_, intact hRBCs were pelleted by low speed centrifugation and the resulting supernatant was transferred to a new microtiter plate. Using the microplate reader, the OD_405nm_ of each supernatant was measured to quantitate the relative concentration of hemoglobin released. All error bars correspond to ± SEM of triplicate experiments.

The cytotoxic effects of PaP1 on neutrophil viability were determined using a previously described method, with modifications (Heselpoth et al., 2019). The HL-60 cell line (ATCC CCL-240) was acquired from the American Type Culture Collection (ATCC). The cells were grown in RPMI 1640 medium (Gibco) supplemented with GlutaMAX (Gibco) and 10% heat-inactivated fetal bovine serum (GE Healthcare). The cells were harvested at 1,500 RPM for 5 min, washed, and resuspended in PBS. Using a 96-well untreated microtiter plate, 1 × 10^6^ HL-60 cells were incubated with PaP1 at final concentrations from 0.5 to 256 μg/ml for 4 h at 37°C with 5% CO_2_. Cells incubated in PBS with or without 0.1% (vol/vol) Triton X-100 were used as positive and negative controls for cytotoxicity, respectively. At the conclusion of the incubation, neutrophil viability was measured using the CellTiter 96 Non-Radioactive Cell Proliferation Assay (Promega). The dye reagent from the assay kit was added to the samples for 4 h at 37°C. Each sample was then mixed with solubilization/stop solution and incubated overnight at 37°C. As a result of living cells converting the tetrazolium dye into a spectrophotometrically detectable formazan product, the relative number of viable neutrophils was quantitated using the microplate reader by measuring the OD_570nm_. All error bars correspond to ± SEM of triplicate experiments.

### Murine Burn Wound Infection Model

All research protocols were approved by The Rockefeller University Institutional Animal Care and Use Committee. A mouse model of full-thickness thermal burns was used in order to evaluate the *in vivo* antibacterial efficacy of PaP1 for preventing and treating burn wound infections. The backs of adult female CD1 mice (6–8 weeks) were shaved with an electric razor and treated with depilatory cream to remove remaining hair. Mice were anesthetized by an intraperitoneal injection of ketamine (1.2 mg/animal) and xylazine (0.25 mg/animal). Following skin disinfection with ethanol, two burn wounds each measuring approximately 1 cm × 1 cm were created by applying two preheated (~90°C) brass rods to opposing sides of a raised dorsal skin fold for 5 s. This burning method has been previously shown to generate non-lethal, full-thickness, third-degree burns (Busch et al., 2000; Dai et al., 2009; Zhang et al., 2014). Immediately after the burn wounds were established, mice were intraperitoneally administered 0.5 ml sterile saline to prevent overt shock. Mice were randomly divided into the subsequent experimental groups in an unbiased manner.

For the prophylactic studies, 50 μl of diH_2_O with or without (untreated controls) 1% (wt/vol) PaP1, 0.02% or 0.2% (wt/vol) mupirocin (Sigma-Aldrich), or 1% PaP1 combined with 0.02% or 0.2% mupirocin, was topically applied to the burn wounds. Following drug absorption (~1 h), a 10 μl suspension containing 10^6^ CFU of mid-log *S. aureus* strain NRS384 (MRSA) was topically applied to the eschar of each burn. At 2 h post-infection, the burn wounds were again treated as previously mentioned above. The mice were euthanized 1 h later, and the burn wounds were excised. Each skin sample was homogenized in 500 μl PBS for 1 min using a Stomacher 80 Biomaster (Seward, Ltd.). Both 100 μl and 10-fold serial dilutions of the homogenate were plated on Mannitol Salt Agar containing 4 μg/ml oxacillin in order to assess bacterial viability. Taking into consideration that both the minimum detectable CFUs per homogenate were five and the average weight of each skin sample was 0.06 g, the resulting limit of detection was approximately 10^2^ CFU/g tissue. Horizontal bars correspond to the geometric mean.

For the treatment studies, a 10 μl suspension containing 10^1^–10^2^ CFU of mid-log *P. aeruginosa* strain NR-51517 (MDR) was applied topically to each burn wound. At 24 h post-infection, mice were anesthetized and treated topically with 50 μl of Aquaphor® with or without (untreated controls) 1% (wt/wt) PaP1, 0.1% or 1% (wt/wt) gentamicin (Fisher Scientific), or 1% PaP1 combined with 0.1% or 1% gentamicin. After 24 h of treatment, mice were euthanized and the skin samples were excised. All skin samples were homogenized in 500 μl PBS for 1 min using a Stomacher 80 Biomaster. Finally, 100 μl and 10-fold serial dilutions of the homogenate were plated on *Pseudomonas* Isolation Agar in order to quantitate surviving bacteria. The limit of detection was approximately 10^2^ CFU/g tissue. Horizontal bars represent the geometric mean.

### Data Analysis

GraphPad Prism 9.0 was used for statistical analysis and constructing figures. The Protein Homology/Analogy Recognition Engine 2.0 (PHYRE2; Kelley et al., 2015) was used for generating predicted structures of the PaP1 derivative peptides.

## Results

### Antipseudomonal Activity and Structural Characteristics of PaP1 Derivative Peptides

**Figure 1:**
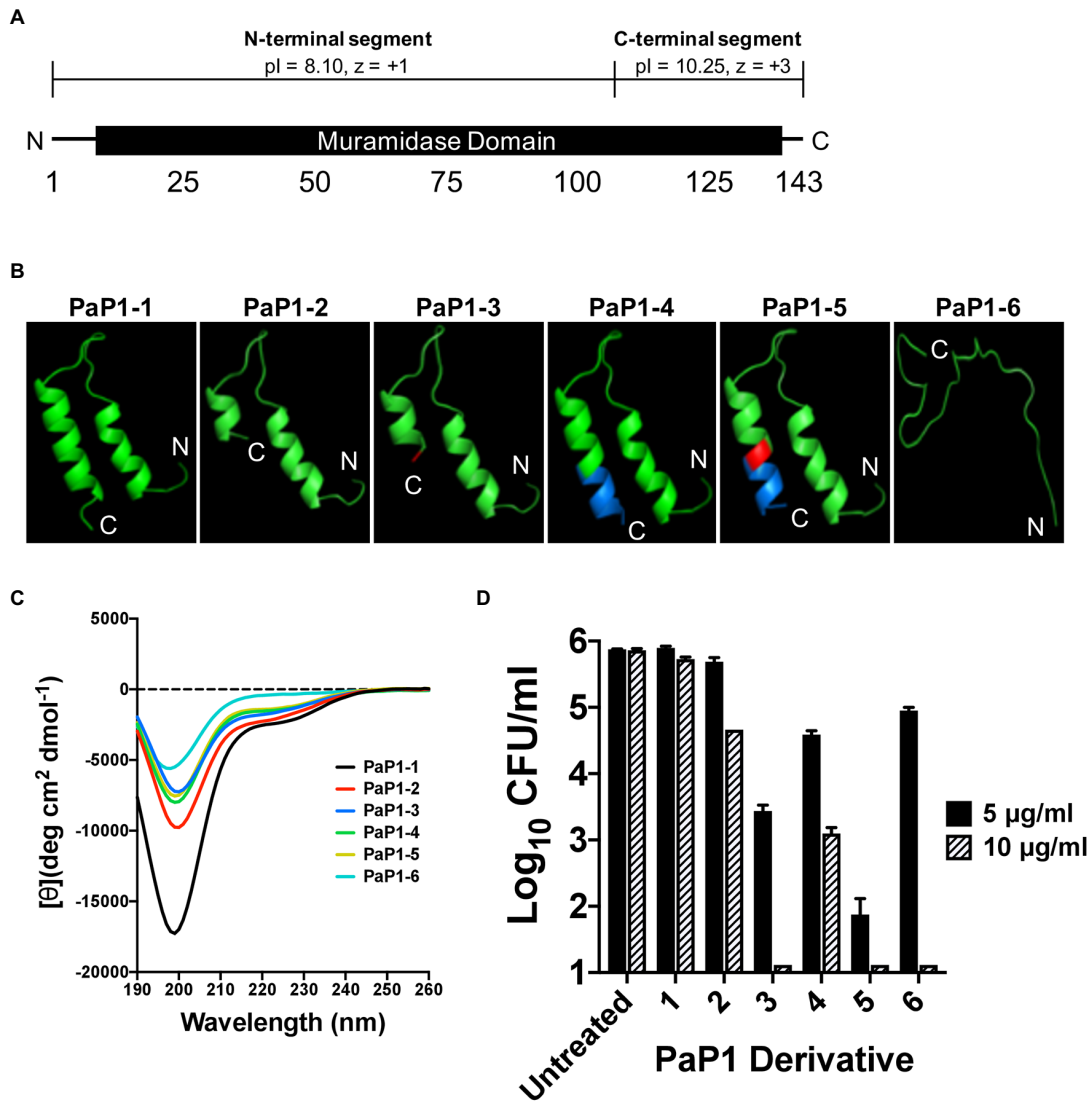
Structural characteristics and antipseudomonal activity of PaP1 derivative peptides. **(A)** The 143 aa *P. aeruginosa* lysin PlyPa01 is predicted to fold as a single globular domain that functions as a muramidase. The cationic properties associated with C-terminal segment of the lysin (aa 103–143; *pI*: 10.25, *z* = +3) are significantly greater than the N-terminal segment (aa 1–102; *pI*: 8.10, *z* = +1). **(B)** The HLH hairpin structures of PaP1-1 to PaP1-5, and the unstructured random coil formed by PaP1-6, were predicted using PHYRE2. Red, A133K mutation and Blue, SQ-8C sequence. **(C)** Far-UV circular dichroism was used to measure the secondary structure composition of each PaP1 derivative peptide at 0.1 mg/ml in 20 mM sodium phosphate, pH 7.0. Spectra were obtained from 190 to 260 nm, with the mean residue ellipticity [θ] (deg cm^2^ dmol^−1^) plotted against the wavelength (nm). **(D)** The bactericidal activity of each PaP1 derivative peptide was measured at either 5 or 10 μg/ml against *P. aeruginosa* strain PAO1 in 20 mM Tris–HCl, pH 7.2, for 1 h at 37°C. Bacterial viability was quantitated following serial dilution and plating. Error bars correspond to ± SEM of triplicate experiments.

The initial peptide candidates evaluated were derived from the *P. aeruginosa* lysin PlyPa01 (accession WP_058157505), which consists of 143 aa that encode a single muramidase domain (aa 7–140; Figure 1A). Similar to the *A. baumannii* lysin PlyF307, PlyPa01 has a putative membrane-acting C-terminal segment (aa 103–143) that has a significantly higher theoretical isoelectric point (*pI*) than the N-terminal segment (aa 1–102; *pI* values of 10.25 and 8.10, respectively), as well as a greater net charge (*z*; *z* values of +3 and +1, respectively; Gasteiger et al., 2005). Considering the full-length PlyPa01 lysin exhibits expanded antibacterial activity toward a collection of GN bacterial pathogens (Raz et al., 2019), we hypothesized that the membrane-acting properties of the C-terminal segment alone could be harnessed as a broad-spectrum antimicrobial.

The C-terminal cationic segment of PlyPa01 was isolated as a peptide, termed PaP1 (*Pseudomonas aeruginosa* Peptide from the PlyPa01 lysin), to assess its potential as an antimicrobial. Several PaP1-related derivative peptides, named PaP1-1 to PaP1-6, were constructed. The amino acid composition of these peptides are as followed: (1) PaP1-1 comprises the full-length C-terminal end of the PlyPa01 lysin (aa 103 to 143); (2) PaP1-2 constitutes a truncated C-terminal end of PlyPa01 (aa 103 to 133); (3) PaP1-3 contains the truncated C-terminal end of PlyPa01 with the terminal alanine residue mutated to a lysine (A133K); (4) PaP1-4 consists of the truncated C-terminal end of PlyPa01 with the C-terminal addition of the SQ-8C sequence from the hepatitis B virus core protein; (5) PaP1-5 encodes the C-terminal truncated end of PlyPa01 with the A133K mutation followed by the SQ-8C sequence; and (6) PaP1-6 comprises a scrambled sequence of PaP1-5 (22.7% sequence identity, 29.5% similarity; Table 2). A133 of PlyPa01_103–133_ was strategically chosen to be mutated to a lysine due to results from a sequence alignment with P307 (data not shown). Despite their shared homology (68% sequence identity, 81% similarity), one notable difference between the sequences is the terminal aa, which is a positively charged lysine for P307 and a neutral-charged alanine for PlyPa01_103–133_. With this in mind, the A133K mutation was introduced to PlyPa01_103–133_ for the purpose of increasing the general cationic properties of the peptide and, more specifically, evaluating the effect a terminal positively charged residue has on antibacterial activity. In general, the physiochemical properties of each PaP1 derivative peptide were strongly cationic (*pI* ≥ 10.25, *z* ≥ +3) and hydrophilic [Grand average of hydropathicity (GRAVY) ≤−0.351].

**Table 2:** Sequence and physiochemical characteristics of PaP1 derivative peptides.

Peptide*^a^*	Amino acid sequence*^b^*	*pI* *^c^*	Charge*^d^*	GRAVY*^e^*
PaP1-1	NAGDYAGAAEQFLRWNKAGGKVLPGLVRRRASERELFLGAA	10.25	+3	−0.351
PaP1-2	NAGDYAGAAEQFLRWNKAGGKVLPGLVRRRA	10.93	+4	−0.506
PaP1-3	NAGDYAGAAEQFLRWNKAGGKVLPGLVRRR**K**	11.00	+5	−0.690
PaP1-4	NAGDYAGAAEQFLRWNKAGGKVLPGLVRRRASQSRESQC	10.28	+4	−0.785
PaP1-5	NAGDYAGAAEQFLRWNKAGGKVLPGLVRRR**K**SQSRESQC	10.43	+5	−0.931
PaP1-6	QYCANDRVGNLWVEAQEGRKPLRSSAKFKGGAQRRSGLA	10.43	+5	−0.931

a *PaP1-5 was renamed as PaP1 for subsequent experiments*.

b *The A133K peptide modification is shown in bold, while the SQ-8C addition is underlined*.

c *Theoretical isoelectric point*.

d *Net charge at pH 7.0*.

e *Grand average of hydropathicity*.

The PHYRE2 structural prediction algorithm revealed that PaP1-1 to PaP1-5 potentially folds into an helix–loop–helix (HLH) hairpin structure, while PaP1-6 (scrambled PaP1-5) is predicted to form a random coil (Figure 1B). Since HLH structures are common motifs found in membrane-active molecules (Ibrahim et al., 2001), far-UV circular dichroism was used to experimentally determine the secondary structure composition of each peptide in sodium phosphate buffer, pH 7.0. For each peptide, the collective far-UV spectra had an ellipticity minima centered around 200 nm (Figure 1C), indicative of random coiled proteins lacking secondary structure. In general, α-helical AMPs are typically unstructured in aqueous solutions but ultimately adopt an amphipathic helical structure upon interacting with its target membrane (Yeaman and Yount, 2003; Pasupuleti et al., 2012).

Using a 1-h killing assay, each PaP1 derivative at 5 and 10 μg/ml was incubated with *P. aeruginosa* to evaluate the effect of each modification on bactericidal activity. The full-length PaP1-1 peptide was ineffective at both concentrations assayed (Figure 1D). Truncating the peptide increased activity, with PaP1-2 reducing bacterial viability 1.2 log_10_-fold at 10 μg/ml. Modifying the truncated peptide with the A133K mutation (PaP1-3) or the C-terminal addition of the SQ-8C sequence (PaP1-4) further enhanced activity. At 10 μg/ml, PaP1-3 decreased bacterial counts below the limit of detection (10 CFU/ml), while PaP1-4 decreased viability 2.8 log_10_-fold. Simultaneously incorporating both the A133K and SQ-8C peptide modifications additively increased the bactericidal potency of the resulting PaP1-5 peptide. Compared to PaP1-3 (2.4 log_10_-fold kill) and PaP1-4 (1.3 log_10_-fold kill) at 5 μg/ml, PaP1-5 reduced pseudomonal viability 4.0 log_10_-fold. Interestingly, scrambling the primary sequence of PaP1-5 yielded a peptide (PaP1-6) that retains bactericidal activity at 10 μg/ml. While this indicates that the aa composition alone is sufficient for antibacterial activity, the defined aa order (i.e., primary sequence) of PaP1-5 is required for optimal killing efficiency. Findings from this comparative analysis showed that derivative peptide PaP1-5 (referred to hereinafter as PaP1) was the most potent and therefore was further evaluated.

### Bactericidal Activity of Additional Lysin-Derived Peptide Candidates

In addition to PlyPa01, several other broad-acting *P. aeruginosa* lysins have been recently described that comprise a cationic C-terminal segment that is potentially membrane-acting (Raz et al., 2019). A sequence alignment with PlyPa01_103–133_ identified the following C-terminal segments from the lysins PlyPa91 (accession CRR10611), PlyPa96 (accession WP_019681133) and PlyPa103 (accession AQZ96894; unpublished) as peptide candidates: PlyPa91_113–144_, PlyPa96_121–162_ and PlyPa103_137–172_. Similar to PaP1, the C-terminal neutral-charged aa of each peptide candidate was mutated to a positively charged lysine and the SQ-8C sequence was added to the C-terminal end. The final modified peptides were labeled as PaP91 (originating from PlyPa91), PaP96 (originating from PlyPa96) and PaP103 (originating from PlyPa103; Table 3). Like the PaP1 derivative peptides, the physiochemical properties associated with the PaP91, PaP96 and PaP103 peptides were highly cationic (*pI* ≥ 9.50, *z* ≥ +4) and hydrophilic (GRAVY ≤−0.934).

**Table 3:** List of peptide candidates originating from broad-acting *Pseudomonas* lysins.

Peptide	Amino acid sequence*^a^*	*pI* *^b^*	Charge*^c^*	GRAVY*^d^*
PlyPa01_103-133_	NAGDYAGAAEQFLRWNKAGGKVLPGLVRRRA	10.93	+4	−0.506
PaP1	NAGDYAGAAEQFLRWNKAGGKVLPGLVRRR**K**SQSRESQC	10.43	+5	−0.931
PlyPa91_113–144_	NAGQPAASWCPELDRWVYAGGKRVQGLVNRRA	10.04	+3	−0.622
PaP91	NAGQPAASWCPELDRWVYAGGKRVQGLVNRR**K**SQSRESQC	9.84	+4	−1.012
PlyPa96_121–162_	NAGNQPAGCRAMLSWRFITRDGKKVDCSTPQPYCSGVWERRQ	9.50	+4	−0.888
PaP96	NAGNQPAGCRAMLSWRFITRDGKKVDCSTPQPYCSGVWERR**K**SQSRESQC	9.50	+5	−1.052
PlyPa103_137–172_	NAGLDINRDGVITKAEAAAKVQAKLDRGLQPQFRRA	10.26	+3	−0.569
PaP103	NAGLDINRDGVITKAEAAAKVQAKLDRGLQPQFRR**K**SQSRESQC	10.02	+4	−0.934

a *The A133K peptide modification is shown in bold, while the SQ-8C addition is underlined*.

b *Theoretical isoelectric point*.

c *Net charge at pH 7.0*.

d *Grand average of hydropathicity*.

**Figure 2:**
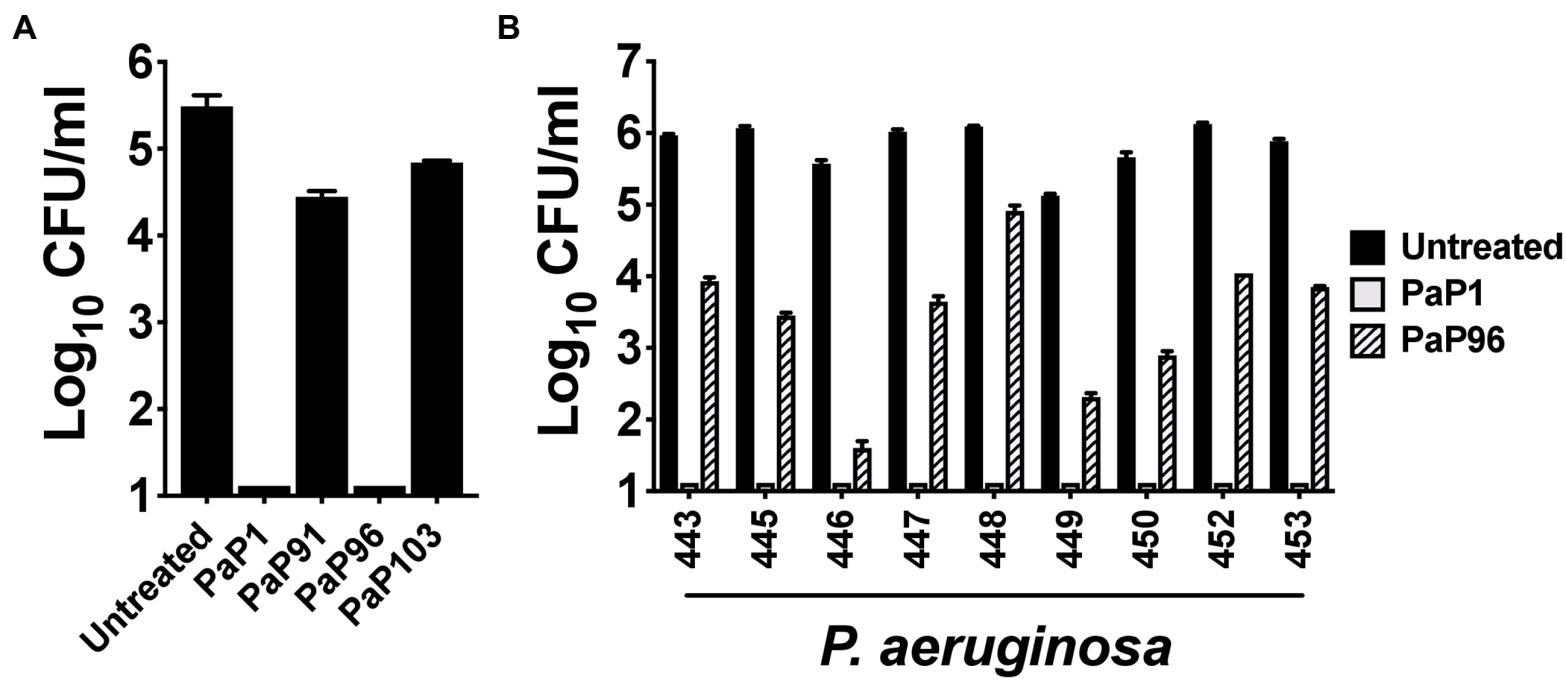
Comparison of the antipseudomonal potency of PaP1 to additional lysin-derived peptide candidates. **(A)** The bactericidal activity of peptide candidates PaP1 (i.e., PaP1-5), PaP91, PaP96 and PaP103 at 10 μg/ml was assayed against *P. aeruginosa* strain PAO1 in 20 mM Tris–HCl, pH 7.2, for 1 h at 37°C. **(B)** The antipseudomonal properties of PaP1 and PaP96 at 10 μg/ml were further measured against nine *P. aeruginosa* clinical isolates. Bacterial viability was quantitated following serial dilution and plating. Error bars correspond to ± SEM of triplicate experiments.

The bactericidal activity of each peptide was initially assayed at 10 μg/ml against the *P. aeruginosa* reference strain PAO1. Like PaP1, PaP96 reduced bacterial viability below the limit of detection (Figure 2A). Alternatively, both PaP91 and PaP103 had only modest antipseudomonal activity (≤1 log_10_-fold kill). The ineffectiveness of PaP91 and PaP103 resulted in the two peptides being eliminated from future studies. The bactericidal properties of PaP1 and PaP96 were further investigated at 10 μg/ml against a collection of nine *P. aeruginosa* clinical isolates. PaP1 reduced the viability of each isolate below the limit of detection, while PaP96 activity was highly variable and strain-dependent (Figure 2B). PaP96 was only bactericidal, which is defined as ≥3 log_10_-fold killing, toward *P. aeruginosa* strains 446 and 449. The collective results from these *in vitro* studies indicate PaP1 has superior antibacterial properties when compared to the other peptide candidates.

### Bactericidal and Biochemical Properties of PaP1

**Figure 3:**
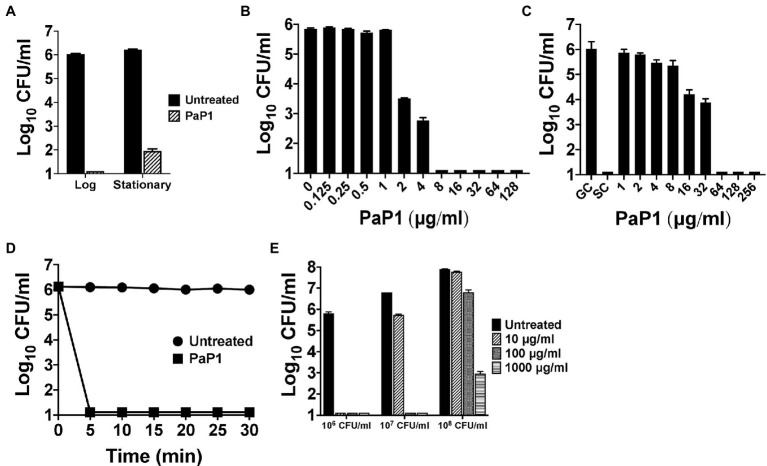
*In vitro* bactericidal properties of PaP1. **(A)** To elucidate the effect bacterial growth phase has on peptide sensitivity, PaP1 at 10 μg/ml was incubated with either mid-log or stationary phase *P. aeruginosa* in 20 mM Tris–HCl, pH 7.2, for 1 h at 37°C. **(B)** Dose–response killing assays were used to evaluate the antipseudomonal potency of PaP1 at concentrations ranging from 0.125 to 128 μg/ml toward planktonic *Pseudomonas* in buffer for 1 h at 37°C. **(C)** The antibiofilm properties of PaP1 were measured *in vitro* against *P. aeruginosa* biofilms grown for 24 h on plastic pegs located on the lid of an MBEC Biofilm Inoculator plate. Peptide concentrations of 1–256 μg/ml were incubated with the mature biofilms in buffer for 2 h at 37°C. Biofilm-state bacteria were eluted from the plastic pegs using a water bath sonicator. GC, growth control and SC, sterility control. **(D)** A time-kill assay was used to analyze the killing kinetics of PaP1 at 10 μg/ml against *P. aeruginosa*. For the 30 min experiment, bacterial viability was determined in buffer every 5 min at 37°C. **(E)** The killing efficiency of PaP1 was evaluated as a function of bacterial concentration. *P. aeruginosa* at 10^6^–10^8^ CFU/ml were treated for 1 h in buffer with PaP1 at concentrations ranging from 10 to 1,000 μg/ml. The CFU/ml concentration of viable bacteria for each experiment was quantitated following serial dilution and plating. Error bars correspond to ± SEM of triplicate experiments.

The bactericidal characteristics of PaP1 were elucidated *in vitro* against *P. aeruginosa* strain PAO1. Bacterial sensitivity to the peptide was initially examined as a function of growth phase. Results from a 1 h killing assay showed that PaP1 was bactericidal regardless of growth phase, although log-phase bacteria were more susceptible than stationary phase (≥5 log_10_-fold vs. 4.3 log_10_-fold kill; Figure 3A). Next, a series of dose–response experiments were performed in order to evaluate the antipseudomonal potency of PaP1. The killing efficiency of the peptide was dose-dependent when treating either planktonic (Figure 3B) or biofilm-state *P. aeruginosa* (Figure 3C), with PaP1 concentrations as low as 2 and 16 μg/ml exhibiting log_10_-fold killing, respectively. Additionally, data obtained from a 30 min time-kill assay revealed the peptide exhibits rapid bacterial killing kinetics. PaP1 at 10 μg/ml sterilized a 10^6^ CFU/ml culture of *P. aeruginosa* within 5 min (Figure 3D). These collective experimental findings show PaP1 is bactericidal in a growth phase-independent manner and kills on contact.

The killing efficiency of PaP1 was subsequently analyzed against varying concentrations of bacteria. Using a 1 h killing assay, PaP1 concentrations from 10 to 1,000 μg/ml were used to treat *P. aeruginosa* cultures at 10^6^–10^8^ CFU/ml. As shown previously, PaP1 at 10 μg/ml was bactericidal toward *Pseudomonas* at 10^6^ CFU/ml (Figure 3E). When increasing the bacterial concentration to 10^7^ and 10^8^ CFU/ml, 10- and 100-fold more peptide was required for bactericidal activity, respectively.

**Figure 4:**
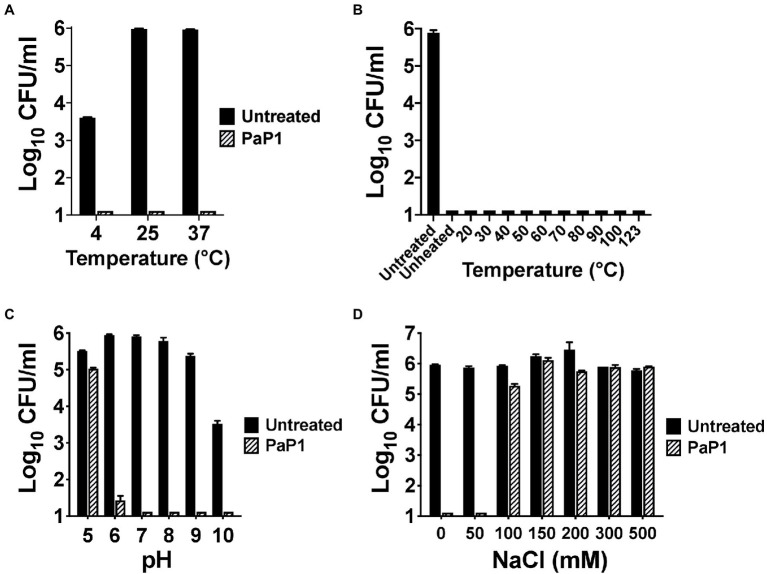
Biochemical characteristics of PaP1. **(A)** The ability of PaP1 to kill bacteria at different temperatures was evaluated by incubating the peptide at 10 μg/ml with *P. aeruginosa* in 20 mM Tris–HCl, pH 7.2, for 1 h at temperatures ranging from 4°C to 37°C. **(B)** The thermal stability of PaP1 was assayed by heating the peptide at 100 μg/ml in PBS, pH 7.4, for 30 min at temperatures ranging from 4°C (unheated control) to 123°C. Following cooling on ice, the heat-treated peptide samples were incubated at 10 μg/ml with *P. aeruginosa* in 20 mM Tris–HCl, pH 7.2, for 1 h at 37°C in order to measure residual activity. **(C)** For measuring the effect pH has on bactericidal activity, PaP1 at 25 μg/ml was incubated with *P. aeruginosa* in 25 mM acetate (pH 5), MES (pH 6), Tris–HCl (pH 7 and 8), CHES (pH 9) and CAPS buffer (pH 10) for 1 h at 37°C. **(D)** The effect salt has on the activity of the peptide was determined by incubating PaP1 at 25 μg/ml with *P. aeruginosa* in 20 mM Tris–HCl, pH 7.2, supplemented with 0–500 mM NaCl for 1 h at 37°C. The CFU/ml concentration of viable bacteria were enumerated following serial dilution and plating. Error bars correspond to ± SEM of triplicate experiments.

Next, the biochemical characteristics of PaP1 were assessed. With regards to temperature dependence for antibacterial activity, the activity of the peptide was unaffected at each temperature tested. PaP1 decreased the number of viable *P. aeruginosa* below the limit of detection at temperatures ranging from 4°C to 37°C (Figure 4A). The thermal stability of the peptide was also studied. Following 30 min of heat treatment from 4°C (unheated control) to 123°C, the residual antipseudomonal activity of PaP1 at 10 μg/ml was measured using a 1 h killing assay. The unheated control and all temperature-treated peptide samples were capable of lowering bacterial viability below the limit of detection, thereby indicating PaP1 is highly thermostable (Figure 4B). This result was not unexpected given the unstructured nature of the peptide in aqueous solutions (Figure 1C).

PaP1 activity was subsequently measured as a function of pH and salt. For the experiments evaluating the pH sensitivity of the peptide, PaP1 reduced the number of viable *Pseudomonas* below the limit of detection in aqueous buffers at pH 7–10 and was bactericidal at pH 6 (Figure 4C). PaP1 activity was inhibited in more acidic environments, with the peptide only capable of lowering bacterial counts 0.5 log_10_-fold at pH 5. In an independent set of experiments assaying PaP1 activity in varying salt concentrations, the peptide decreased *P. aeruginosa* viability below the limit of detection in NaCl concentrations less than 100 mM (Figure 4D). However, PaP1 lacked bactericidal activity in the presence of ≥100 mM NaCl.

### Activity in Serum and Lung Surfactant

**Figure 5:**
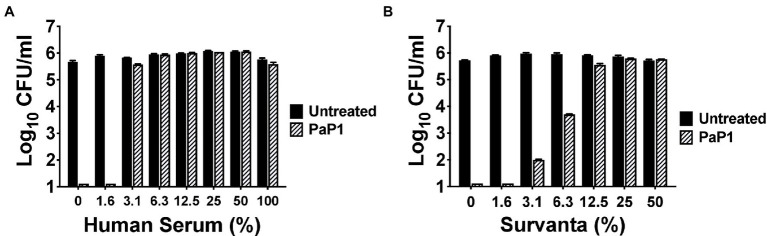
Bacterial killing by PaP1 in the presence of serum and lung surfactant. The sensitivity of PaP1 activity to HuS and lung surfactant was assessed by treating *P. aeruginosa* with the peptide at 25 μg/ml in either **(A)** 0–100% inactivated HuS or **(B)** 0–50% beractant (Survanta) for 1 h at 37°C, with 20 mM Tris–HCl, pH 7.2, being used as the diluent. Bacterial viability was quantitated following serial dilution and plating. Error bars correspond to ± SEM of triplicate experiments.

*P. aeruginosa* is one of the predominant microorganisms that cause nosocomial bloodstream infections (BSIs), with mortality rates exceeding 20% (Micek et al., 2005). To evaluate the potential use of the peptide for treating systemic infections, the *in vitro* antipseudomonal activity of PaP1 was measured in HuS. Results from a 1 h killing assay revealed that peptide activity at 25 μg/ml was hindered by serum components, with low concentrations of HuS (≥3.1%) being inhibitory (Figure 5A). Therefore, in its current form, PaP1 would not be a suitable therapeutic option for treating BSIs.

*P. aeruginosa* is also notably the most common GN pathogen that causes nosocomial pneumonia, a disease with a mortality rate ranging from 13% to 50% (Melsen et al., 2013; Nathwani et al., 2014; Kalil et al., 2016; Kizny Gordon et al., 2017). The surface of the lungs is covered in lung surfactant, which is a mixture of phospholipids and proteins produced by type II alveolar cells used to lower surface tension (Daniels and Orgeig, 2003; Bernhard, 2016). As such, we analyzed the antibacterial activity of PaP1 in this complex environment to simulate conditions observed in the lungs. More specifically, *P. aeruginosa* were treated with the peptide in beractant (Survanta), which is a modified bovine lung surfactant that mimics the composition and surface–tension-lowering properties of natural lung surfactant. PaP1 exhibited log_10_-fold killing of *Pseudomonas* in Survanta concentrations up to 6.3% and was inhibited at concentrations ≥12.5% (Figure 5B). Similar results were obtained when testing the peptide in human bronchoalveolar lavage (BAL) fluid (data not shown). In terms of therapeutic applicability, these findings indicate PaP1 would be more effective for topical applications.

### Antibacterial Activity Range

**Figure 6:**
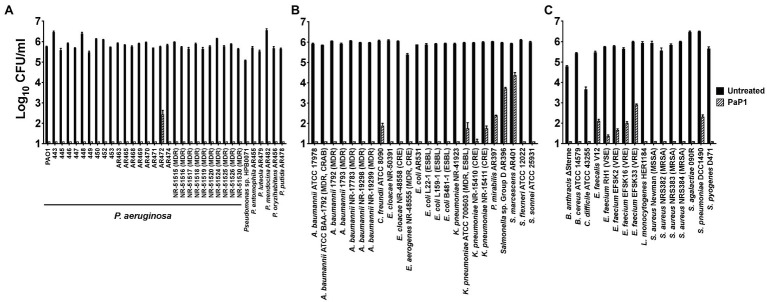
Antibacterial activity range of PaP1. The antibacterial activity of PaP1 was analyzed against **(A)** *Pseudomonas*, **(B)** non-pseudomonal GN or **(C)** GP bacterial species by treating the log-phase bacteria with peptide at 25 μg/ml in 20 mM Tris–HCl, pH 7.2, for 1 h at 37°C. The CFU/ml concentration of surviving bacteria was enumerated following serial dilution and plating. Error bars correspond to ± SEM of triplicate experiments.

The antibacterial activity of PaP1 at 25 μg/ml was evaluated *in vitro* against an extensive number of GP and GN bacterial pathogens using a series of 1 h killing assays (Figure 6). The peptide was initially tested against 28 *P. aeruginosa* strains, including ten MDR clinical isolates, and six additional *Pseudomonas* species. PaP1 was bactericidal against all *Pseudomonas* strains tested (Figure 6A). With the exception of *P. aeruginosa* strain AR472, PaP1 reduced the number of viable bacteria below the limit of detection.

These studies were expanded to other GN bacterial pathogens, such as *A. baumannii*, *P. mirabilis*, *Serratia marcescens*, and Enterobacteriaceae, such as *Citrobacter freundii*, *Enterobacter* spp., *E. coli*, *Klebsiella pneumoniae*, *Salmonella* and *Shigella* spp. Other than *S. marcescens* and *Salmonella*, PaP1 was bactericidal against all GN species investigated, including antibiotic-resistant strains (Figure 6B). Notable antibiotic-resistant GN bacteria evaluated include MDR and carbapenem-resistant (CRAB) *A. baumannii*, extended-spectrum beta-lactamase (ESBL) *E. coli*, and carbapenem-resistant Enterobacteriaceae (CRE), such as *Enterobacter aerogenes*, *Enterobacter cloacae* and *K. pneumoniae*. For *S. marcescens* and *Salmonella*, the peptide still exhibited log_10_-fold killing of both bacterial species.

PaP1 activity was subsequently assayed against clinically relevant GP bacterial pathogens, such as *Bacillus anthracis*, *C. difficile*, *Enterococcus* spp., *Listeria monocytogenes*, *S. aureus* and *Streptococcus* spp. PaP1 was bactericidal against all GP bacteria examined, including antibiotic-resistant strains like vancomycin-resistant *Enterococcus* (VRE) and methicillin-resistant *S. aureus* (MRSA; Figure 6C). With the exception of enterococcus and pneumococcus, both of which were killed multi-log_10_-fold, the peptide reduced bacterial viability below the limit of detection (~≥5 log_10_-fold killing), collectively indicating that PaP1 is a broad-spectrum antimicrobial.

### Bactericidal Activity Toward a Polymicrobial Bacterial Population

**Figure 7:**
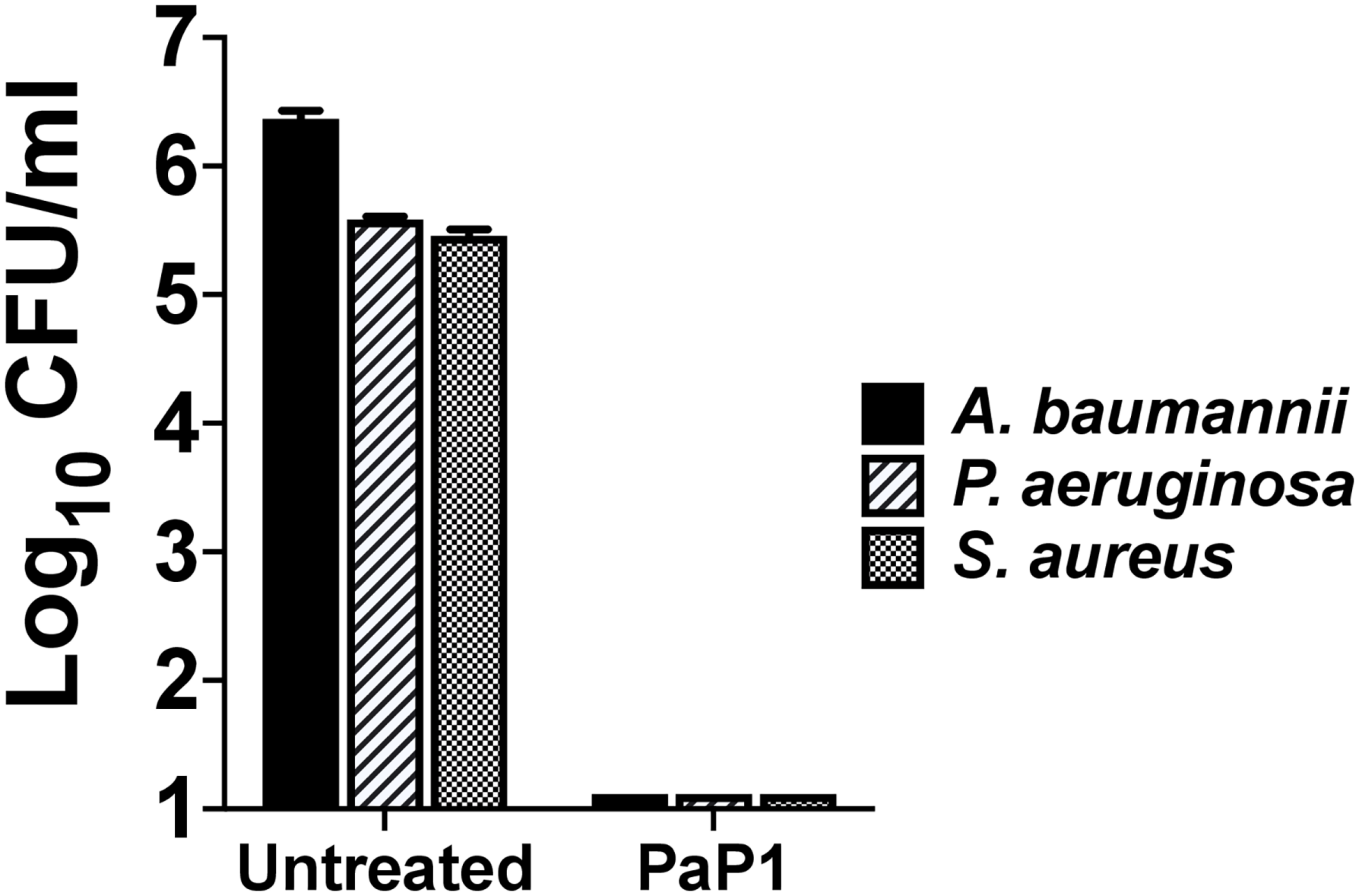
Bactericidal activity of PaP1 toward a polymicrobial culture of antibiotic-resistant human wound isolates. The effectiveness of PaP1 in a polymicrobial setting was evaluated by simultaneously treating mid-log *A. baumannii* strain NR-17783 (MDR) *P. aeruginosa* strain NR-17783 (MDR), and *S. aureus* strain NRS384 (MRSA) with the peptide at 50 μg/ml in 20 mM Tris–HCl, pH 7.2, for 1 h at 37°C. The bacteria were serial diluted and plated on selective agar in order to assess viability. Error bars correspond to ± SEM of triplicate experiments.

Seeing as PaP1 displayed broad-spectrum *in vitro* activity against numerous bacterial cultures containing a single species of bacteria, the effectiveness of the peptide was analyzed next against a polymicrobial culture. The mixture of bacteria tested consisted of MRSA and MDR strains of *A. baumannii* and *P. aeruginosa*, each of which was isolated from human wounds. These three bacterial species are among the most common types that cause burn wound infections (Chen et al., 2020). A polymicrobial culture comprising ~10^5^–10^6^ CFU/ml of each bacterial species was treated with PaP1 at 50 μg/ml. Following treatment for 1 h at 37°C, PaP1 reduced the number of each species below the limit of detection (Figure 7). Based on these findings, PaP1 is expected to retain potent bactericidal activity in polymicrobial environments.

### Antibacterial Mode of Action

**Figure 8:**
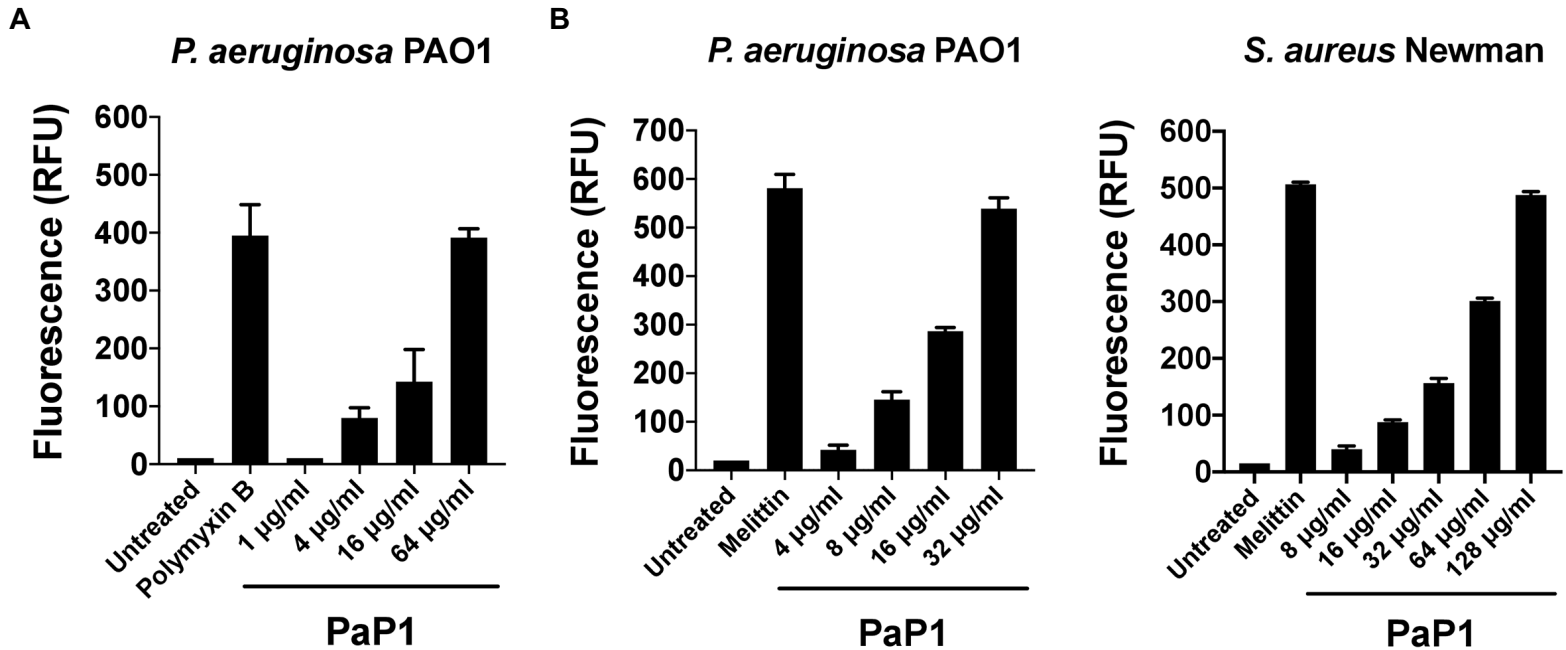
Microbial membrane permeabilization caused by PaP1. The proposed membrane-acting antibacterial mode of action utilized by PaP1 was evaluated using assays that measure membrane integrity. **(A)** The effect of increasing concentrations of PaP1 on the permeability of the bacterial OM was quantitated using the NPN uptake assay. *P. aeruginosa* were treated with peptide concentrations from 1 to 64 μg/ml in 20 mM Tris–HCl, pH 7.2, containing 25 μM of the hydrophobic fluorophore NPN for 1 h at 37°C. NPN fluorescence, which increases upon integration into the OM, was measured using an excitation wavelength at 350 nm and emission at 420 nm. Polymyxin B at 2 μg/ml was used as a positive control for OM permeabilization. **(B)** The structural integrity of the CM for either *P. aeruginosa* (left) or *S. aureus* (right) was elucidated in the presence of PaP1 at concentrations from either 4 to 32 μg/ml (*P. aeruginosa*) or 8 to 128 μg/ml (*S. aureus*) using the SYTOX Green uptake assay. Bacteria were initially pretreated with 5 μM of SYTOX Green dye and then subsequently treated with PaP1 in 20 mM Tris–HCl, pH 7.2, for 1 h at 37°C. Dye fluorescence, which increases after the dye bypasses compromised membranes to bind DNA, was measured using an excitation wavelength at 485 nm and emission at 520 nm. Melittin at 10 μg/ml was used as a positive control for CM disruption. Data were normalized to the untreated controls. Error bars correspond to ± SEM of triplicate experiments. RFU, relative fluorescence units.

Considering that the C-terminal cationic segment of PlyPa01 is putatively membrane-acting, we hypothesized that PaP1 kills bacteria *via* membrane disruption. To confirm this, the integrity of the bacterial OM and/or CM were assayed following treatment with PaP1. First, the NPN uptake assay was used to measure the permeability of the OM of *P. aeruginosa* in the presence of increasing concentrations of peptide. The hydrophobic fluorophore NPN, which is generally excluded from the bacterial cell, is incorporated into the phospholipid bilayer of the OM when damaged, resulting in increased fluorescence intensity. The membrane-acting antibiotic polymyxin B was used as a positive control for OM permeabilization. Data from these experiments suggest the PaP1 peptide increases the permeability of the OM in a dose-dependent manner (Figure 8A).

The integrity of the CM was assayed for PaP1-treated *P. aeruginosa* and *S. aureus* using the SYTOX Green uptake assay. SYTOX Green is a membrane impermeant, DNA binding dye. Although blocked by intact membranes, the dye easily penetrates through compromised membranes to interact with DNA, as indicated by a significant increase in fluorescence. The pore-forming cationic peptide melittin, the active compound in bee venom known to damage the CM of bacteria, served as a positive control (Raghuraman and Chattopadhyay, 2007). Treatment with PaP1 disrupted the CM of both *P. aeruginosa* (Figure 8B, left) and *S. aureus* (Figure 8B, right) in a concentration-dependent manner. Four-fold more peptide was required to obtain a similar degree of CM damage for *S. aureus* when compared to *P. aeruginosa*. Altogether, these results strongly support the hypothesis that PaP1 kills bacteria *via* membrane disruption.

### Cytotoxicity Toward Human Cells

**Figure 9:**
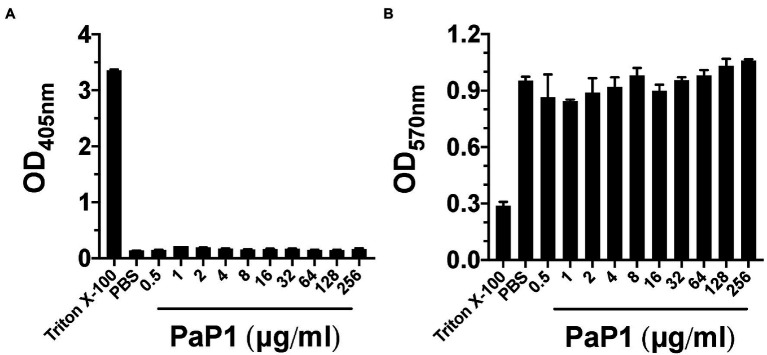
PaP1 cytotoxicity toward human cells. **(A)** hRBCs from healthy donors were incubated with PaP1 concentrations from 0.5 to 256 μg/ml in PBS for 4 h at 37°C in 5% CO_2_. Following the removal of intact hRBCs, the relative concentration of hemoglobin released in each sample supernatant was quantitated by measuring the absorbance at OD_405nm_. As controls for hemolysis, hRBCs were incubated in buffer with (positive control) or without 0.1% Triton X-100 (negative control). **(B)** HL-60 neutrophils were incubated with PaP1 concentrations from 0.5 to 256 μg/ml in PBS for 4 h at 37°C in 5% CO_2_. Next, the tetrazolium substrate was added for 4 h. Each sample was then mixed with solubilization/stop solution and incubated overnight at 37°C. The absorbance at OD_570nm_ was measured in order to quantitate the relative concentration of formazan product generated by live cells. As controls for cytotoxicity, neutrophils were incubated in buffer with (positive control) or without 0.1% Triton X-100 (negative control). Error bars correspond to ± SEM of triplicate experiments.

To determine the effect of PaP1 on mammalian cells, the peptide was incubated with hRBCs at concentrations up to 256 μg/ml in PBS for 4 h. Hemolysis, as a function of hemoglobin release, was measured spectrophotometrically following the removal of intact hRBCs. Triton X-100 and buffer served as respective positive and negative controls for hemolysis. Similar to the buffer-treated cells, hRBCs were unaffected by PaP1 at all concentrations tested (Figure 9A).

Findings from the hemolytic assay were further validated upon measuring the cytotoxic effect PaP1 has toward neutrophils. HL-60 neutrophils were incubated with PaP1 at concentrations up to 256 μg/ml in PBS for 4 h. Using a cell proliferation assay, the relative number of viable cells were quantitated due to their ability to convert tetrazolium into a spectrophotometrically detectable formazan product. Triton X-100 and buffer represented positive and negative controls for cytotoxicity, respectively. Unlike the Triton X-100 samples, the relative number of viable neutrophils observed in the buffer- and peptide-treated samples were highly comparable across all peptide concentrations tested (Figure 9B). The lack of cytotoxicity toward the human cells tested suggests that PaP1 exhibits selective activity toward prokaryotic membranes only.

### *In vivo* Antibacterial Efficacy of PaP1 using a Murine Burn Wound Model

**Figure 10:**
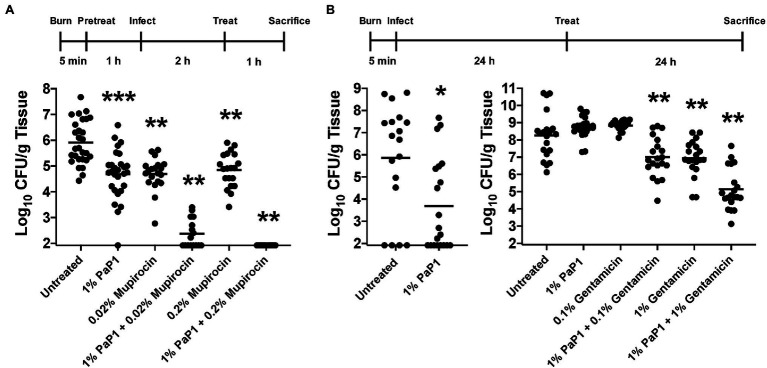
Bacterial clearance by PaP1 in a murine burn wound infection model. **(A)** For the *in vivo* prophylactic studies, third-degree burns on mice were pretreated with either diH_2_O only (*n* = 28) or diH_2_O supplemented with 1% PaP1 (*n* = 28), 0.2% (*n* = 20) or 0.2% mupirocin (*n* = 20), or a combination therapy comprising 1% PaP1 with 0.02% (*n* = 16) or 0.2% mupirocin (*n* = 16). After 1 h, burn wounds were subsequently infected with 10^6^ CFU of MRSA and then treated 2 h later with a single drug dose. At 3 h post-infection, burn wounds were processed in order to determine the CFU/g tissue concentration of viable bacteria. Statistically significant differences were noted between the untreated control and either 1% PaP1 (*p* = 0.001), 0.02% mupirocin (*p* = 0.001), 1% PaP1 with 0.02% mupirocin (*p* = 0.002), 0.2% mupirocin (*p* = 0.001), or 1% PaP1 with 0.2% mupirocin (*p* = 0.002) using an ordinary one-way ANOVA, multiple comparisons, uncorrected Fisher LSD test. **(B)** For the *in vivo* treatment studies, third-degree burns on mice were infected with 10^1^–10^2^ CFU of MDR *P. aeruginosa* (left). At 24 h post-infection, the infected wounds were treated with either Aquaphor only (*n* = 18) or Aquaphor formulated with 1% PaP1 (*n* = 20). Statistically significant differences were shown between the untreated control and 1% PaP1 (*p* = 0.011) using a Mann–Whitney test. (right) For studies utilizing the combination therapy consisting of PaP1 and gentamicin, burn wounds colonized with about 100-fold more bacteria were treated with Aquaphor only (*n* = 20) or Aquaphor formulated with either 1% PaP1 (*n* = 23), 0.1% (*n* = 20) or 1% gentamicin (*n* = 20), or a combination therapy of 1% PaP1 with 0.1% (*n* = 20) or 1% gentamicin (*n* = 20). All burn wounds were processed at 24 h post-treatment in order to determine the CFU/g tissue concentration of viable bacteria. Statistically significant differences were highlighted between the untreated control and either 1% PaP1 with 0.1% gentamicin (*p* = 0.005), 1% gentamicin (*p* = 0.005), or 1% PaP1 with 1% gentamicin (*p* = 0.005) using an ordinary one-way ANOVA, multiple comparisons, uncorrected Fisher LSD test. The limit of detection was approximately 10^2^ CFU/g tissue. Horizontal lines represent the geometric mean. Significance levels are depicted as followed: *p < 0.05, **p ≤ 0.01, ***p ≤ 0.001.

Results from our *in vitro* studies show that PaP1 may be optimally suited for topical applications, such as treating burn wound infections. While these infections may be polymicrobial, high priority bacterial pathogens, such as *S. aureus* and *P. aeruginosa*, tend to predominate (Pastar et al., 2013). To this end, the antistaphylococcal and antipseudomonal efficacy of PaP1 was evaluated *in vivo* using a murine burn wound model of disease. In the first set of experiments (Prophylactic model), 1% (wt/vol) PaP1, 0.02% or 0.2% (wt/vol) mupirocin, or a combination of the two drugs were administered topically in diH_2_O as a prophylactic to murine third-degree burn wounds. diH_2_O was applied to the wounds of the untreated controls. One hour after drug application, the wounds were infected with 10^6^ CFU of MRSA strain NRS384 (i.e., strain USA300; human wound isolate), followed by a second dose of drugs 2 h post-infection. Mice were then sacrificed 3 h post-infection, and wounds were excised for bacterial counts. Compared to the untreated control (~10^6^ CFU/g tissue), PaP1 and mupirocin alone reduced MRSA viability 1.1–1.2 log_10_-fold (Figure 10A). A significantly more pronounced antistaphylococcal effect was observed when the two drugs were used in combination. The combination of 1% PaP1 and 0.02% mupirocin reduced bacterial counts 3.5 log_10_-fold, whereas 1% PaP1 with 0.2% mupirocin decreased MRSA viability below the limit of detection (≥4 log_10_-fold kill). Notably, the two concentrations of mupirocin evaluated in this study were 10- and 100-fold lower than the 2% concentration used clinically for topically treating chronic wound infections caused by *S. aureus* (Lipsky and Hoey, 2009).

In a related set of experiments (Therapeutic model), the therapeutic potential of PaP1 was assessed with regards to treating an established burn wound infection caused by *P. aeruginosa*. For these animal studies, murine third-degree burns were initially infected with 10^1^–10^2^ CFU of MDR *P. aeruginosa* strain NR-51517 (human wound isolate). At 24 h post-infection, each wound containing an established bacterial infection was treated topically with a single dose of either Aquaphor only (untreated controls) or Aquaphor formulated with 1% (wt/wt) PaP1. Bacterial counts in the control wounds 24 h post-treatment were ~10^6^ CFU/g tissue (Figure 10B, left). Treatment with PaP1 in Aquaphor reduced bacterial counts 2.2 log_10_-fold.

Subsequent *in vivo* experiments were performed in order to investigate the efficacy of a PaP1 and gentamicin combination therapy for treating established *P. aeruginosa* burn wound infections. In the previous studies, 1% PaP1 lowered bacterial viability below the limit of detection for 40% (8/20) of the skin samples (Figure 10B, left). To decrease the sensitivity of *Pseudomonas* to the peptide in order to assess the effectiveness of the combination therapy, the number of bacteria colonizing each murine burn wound was increased ~100-fold. Murine third-degree burn wounds infected with MDR *P. aeruginosa* were then treated topically 24 h post-infection with either Aquaphor only (untreated controls) or Aquaphor formulated with 1% (wt/wt) PaP1, 0.1% or 1% (wt/wt) gentamicin, or a combination of the two drugs. Bacterial counts observed for wounds treated with a 1% PaP1 or 0.1% gentamicin monotherapy were comparable to those observed for the Aquaphor-treated controls (~10^8^ CFU/g tissue), while 1% gentamicin decreased the bacterial load 1.3 log_10_-fold (Figure 10B, right). A combination therapy was more effective than using a monotherapy. Treatment using 1% PaP1 with 0.1% gentamicin reduced pseudomonal viability 1.3 log_10_-fold, whereas 1% PaP1 combined with 1% gentamicin exhibited 3.1 log_10_-fold killing. Data obtained from these studies indicate that PaP1 can potentially be developed and formulated as an effective prophylactic and/or therapeutic for both GP and GN burn wound infections.

## Discussion

Bacterial infections are the leading cause of mortality in patients with burn wounds, with 75% of all burn-related deaths resulting from infection (Maslova et al., 2021). Treating these infections is further complicated by the increasing prevalence of antibiotic-resistant strains. For example, a single-center study from 2008 to 2012 reported that 33.8%, 90.8% and 82% of *Pseudomonas* spp., *A. baumannii* and *S. aureus* isolates causing hospital-acquired infections were MDR, respectively (Weber et al., 2014). To overcome this challenge, we propose using membrane-acting cationic peptides originating from highly evolved GN phage lysins as alternative therapeutics for treating topical bacterial infections. This strategy is formulated in accordance with previous findings of a lysin-derived peptide, P307_SQ-8C_, exhibiting log_10_-fold killing of MDR *A. baumannii* in a skin infection model of disease (Thandar et al., 2016). These cationic peptides can theoretically be engineered as an effective standalone or adjunctive therapy that broadly eliminates prominent bacterial pathogens from the skin without harming bystander eukaryotic cells.

The lead peptide candidate from our studies, PaP1, originates from the PlyPa01 lysin of *P. aeruginosa* (Figure 1A). This peptide was constructed with three modifications: (a) the C-terminal membrane-acting segment of PlyPa01 was isolated and truncated to improve its cationic properties, (b) the resulting C-terminal neutral-charged alanine residue was mutated to a positively charged lysine to increase the net charge, and (c) the SQ-8C sequence from the hepatitis B virus core protein was added to the C-terminal end, a modification that was shown previously to enhance the antibacterial potency of such peptides (Table 2; Thandar et al., 2016). Each independent alteration incrementally increased the killing efficiency of the peptide toward *P. aeruginosa* (Figure 1D) and ultimately other pathogens (Figure 6).

Results from the NPN and SYTOX Green uptake assays revealed the general antibacterial mode of action used by PaP1 is membrane disruption (Figure 8). This is further supported by the understanding that PaP1 kills bacteria on contact (Figure 3D). In general, membrane-acting AMPs rapidly kill bacteria, whereas AMPs and antibiotics targeting intracellular components exhibit slower killing kinetics (Schneider et al., 2010). Similar to cationic AMPs, PaP1 was unstructured in aqueous solution (Figure 1C), but is expected to fold into an amphipathic HLH structure upon interacting with the bacterial membrane due to the hydrophobic residues (Figure 1B). This would consequently allow the peptide to integrate into and/or traverse the membrane. Following the initial electrostatic interaction with the bacterial surface, it remains elusive how the peptide mechanistically damages the bacterial membrane. For GN bacteria, some cationic peptides bind surface LPS and displace stabilizing divalent cations that are essential for OM structural integrity (Friedrich et al., 2000). These events permeabilize the OM and allow the peptide to travel through the thin peptidoglycan layer to consequently access the CM. For GP bacteria, cationic peptides can diffuse through the nano-sized pores of the thicker peptidoglycan in order to electrostatically interact the anionic lipoteichoic acids and phospholipids located on the CM (Meroueh et al., 2006; Omardien et al., 2016). Following accumulation on the surface, cationic peptides generally disrupt the CM by either (a) forming barrel-like bundles in the membrane to generate amphipathic pores (barrel-stave model), (b) generating carpet-like clusters aligned in parallel on the surface, causing the membrane to collapse into micelle-like structures (carpet model), or (c) creating peptide–lipid lined pores (toroidal pore model; Seo et al., 2012). The linear correlation between bacterial concentration and the antibacterial potency of PaP1 could indicate that the interaction between the peptide and bacterial membrane is irreversible (Figure 3E).

PaP1 displayed *in vitro* bactericidal activity toward numerous bacterial pathogens of clinical importance, including monospecies (Figure 6) and polymicrobial populations (Figure 7) constituting antibiotic-resistant isolates. Importantly, each of the ESKAPE pathogens (*Enterococcus faecium*, *S. aureus*, *K. pneumoniae*, *A. baumannii*, *P. aeruginosa*, and *Enterobacter* spp.) were highly sensitive to the peptide. Planktonic and biofilm bacteria were susceptible to PaP1, although an eight-fold higher concentration was required for the latter to achieve comparable killing efficiency (Figures 3B,C). Nonetheless, the broad-spectrum bactericidal activity of PaP1 coupled with its ability to kill biofilm-state bacteria implicates the peptide as a therapeutic for non-systemic monospecies and mixed-species biofilms. Moreover, the peptide could also potentially be used in healthcare settings for disinfecting surgical devices prior to implantation.

For PaP1, the absence of cytotoxicity toward eukaryotic cells (Figure 9) could be explained by differences in membrane composition when compared to prokaryotes. Bacterial membranes are decorated with anionic phospholipids, while eukaryotic membranes contain cholesterol and zwitterionic phospholipids (Glukhov et al., 2005). The cationic properties of PaP1 could permit selective electrostatic interactions with the negatively charged surface of microbial membranes only. The hypothesis that adsorption of PaP1 to the bacterial membrane appears to be electrostatically driven is supported by the direct correlation between the charge of the peptide and antibacterial activity (Table 2; Figure 1D), as well as salt sensitivity (Figure 4D).

As observed with PaP1, the bactericidal activity of AMPs can be inhibited by the presence of salt and serum. Salt can influence the structure of the peptides and their ability to interact with the anionic bacterial cell surface (Javadpour and Barkley, 1997). Moreover, cationic AMPs generally exhibit high affinity for serum proteins, decreasing the concentration of peptide available for eliminating bacteria (Li et al., 2017). Therefore, it was not unexpected to observe salt (Figure 4D) and serum sensitivity (Figure 5A) associated with PaP1. However, this limitation may be overcome by introducing additional modifications to the peptide. For instance, synthetic α-helical peptides have been engineered to be salt-insensitive by optimizing the amphipathicity and stability of the α-helix (Friedrich et al., 1999; Park et al., 2004). Also, end-tagging AMPs with short hydrophobic oligopeptides have been shown to increase antibacterial activity in physiological salt and serum (Pasupuleti et al., 2009; Schmidtchen et al., 2009). Preliminary findings indicate that adding the WWWWW oligopeptide to the C-terminal end of PaP1 decreases salt sensitivity, with the modified peptide capable of killing *P. aeruginosa* at physiological salt concentrations (data not shown).

One of the greatest challenges in treating bacterial infections in burn units is their resistance to small molecule antibiotics, which is further complicated by the lack of new antimicrobial agents currently being developed. In addition to *A. baumannii*, the most common microorganisms isolated from burn wound infections are *S. aureus* and *P. aeruginosa* (van Langeveld et al., 2017). When applied to a full-thickness murine burn wound, the prophylactic use of PaP1 as a standalone treatment decreased MRSA colonization (Figure 10A). However, the antistaphylococcal effect of the peptide was amplified when used in combination with mupirocin. Supplementing 1% PaP1 with 0.02% mupirocin was bactericidal and lowered the bacterial concentration on the skin more than 2 log_10_-fold below the critical concentration threshold of 10^5^ CFU/g tissue, which is estimated to be the microbial load required to establish both acute and chronic skin infections (Bowler et al., 2001). Furthermore, combining 1% PaP1 with 0.2% mupirocin prevented any detectable MRSA from colonizing the burn wound.

A topical therapy consisting of PaP1 with or without gentamicin was also effective at treating established burn wound infections consisting of MDR *P. aeruginosa*. A single dose of 1% PaP1 reduced pseudomonal viability in the burn wounds from 10^6^ to less than 10^4^ CFU/g tissue (Figure 10B, left). In a subsequent series of experiments evaluating the efficacy of a PaP1 and gentamicin combination therapy, using bacterial concentrations significantly higher than those normally observed in burn wounds (~10^8^ CFU/g tissue) resulted in monotherapies consisting of either 1% PaP1 or 0.1% gentamicin being largely ineffective (Figure 10B, right). As shown *in vitro*, the sensitivity of *P. aeruginosa* to PaP1 is directly influenced by the bacterial concentration (Figure 3E). With 100-fold more bacteria colonizing the eschar in these studies, higher peptide concentrations would be required to obtain the same degree of killing efficiency as observed in the initial treatment experiments. An adjunctive therapy comprising PaP1 with a standard of care antibiotic can serve as an alternative strategy to increasing peptide concentration. This was exemplified by the antibacterial effect observed when applying 1% PaP1 and 0.1% gentamicin in combination to the established burn wound infections. A similar observation was made when increasing the gentamicin concentration. Compared to 1% gentamicin alone (1.3 log_10_-fold kill), combining 1% PaP1 with 1% gentamicin significantly improved antipseudomonal potency (3.1 log_10_-fold kill). Thus, the combination of PaP1 with standard of care antibiotics improves antibacterial efficacy and can prevent toxicity and other side effects associated with high doses of single drugs.

When formulated with PaP1, the increased *in vivo* potency of mupirocin and gentamicin, two antibiotics currently used for topical antibacterial treatment (Lipsky and Hoey, 2009; Thapa et al., 2021), could be the result of the peptide permeabilizing the bacterial membrane. This would stimulate the rapid intracellular accumulation of the antibiotic and easier access to their cytosolic targets (isoleucyl-tRNA synthetase for mupirocin, 30S ribosomal subunit for gentamicin). Moreover, since these antimicrobials employ two unique antibacterial modes of action, membrane disruption for PaP1 and protein synthesis inhibition for the antibiotics, we predict a low frequency of resistance formation to either antimicrobial when used in combination.

Optimization of both dosage regimen and drug delivery could improve the antibacterial potency of a PaP1 monotherapy. For example, to simulate other treatment protocols implemented in healthcare settings, multiple doses of PaP1 could be topically applied to the infected burn wounds instead of the single treatment dose used here. Additionally, optimal formulations of PaP1 (i.e., by spray, gel or cream) would enable the efficient delivery and retention of the peptide at the site of infection.

In summary, we engineered a lysin-derived cationic peptide, termed PaP1, with broad-spectrum antibacterial activity against prominent bacterial pathogens, including both the ESKAPE pathogens and those found predominantly in burn wound infections. PaP1 displayed rapid *in vitro* bactericidal activity toward planktonic and biofilm-state bacteria, with the peptide killing bacteria on contact through membrane disruption. Although membrane-acting, the peptide selectively targets prokaryotic membranes and thus does not harm human cells. Considering the inhibitory effects of serum, lung surfactant, and high salt concentrations, the therapeutic use of the peptide is best suited for topical applications. Topical PaP1 was quite effective at reducing colonization of MRSA and established MDR *P. aeruginosa* infections in murine burn wound models of disease and improved the standard of care when utilized as an adjunctive therapy with antibiotics. Although still in the preclinical development stage, these initial collective results substantiate PaP1 as a potential standalone or adjunctive therapeutic for both the prevention and treatment of polymicrobial skin infections.

## Data Availability Statement

The original contributions presented in the study are included in the article/supplementary material; further inquiries can be directed to the corresponding author.

## Ethics Statement

The animal study was reviewed and approved by The Rockefeller University Institutional Animal Care and Use Committee.

## Author Contributions

RH, CE, and VF conceived and designed different aspects of the research project and interpreted data obtained from experiments. RH and CE performed the experiments. RH wrote the manuscript. CE and VF critically edited the manuscript. VF supervised the project. All authors contributed to the article and approved the submitted version.

## Funding

This research was funded by laboratory funds from The Rockefeller University.

## Conflict of Interest

RH, CE, and VF recently filed a provisional patent for the technology described in the paper.

## Publisher’s Note

All claims expressed in this article are solely those of the authors and do not necessarily represent those of their affiliated organizations, or those of the publisher, the editors and the reviewers. Any product that may be evaluated in this article, or claim that may be made by its manufacturer, is not guaranteed or endorsed by the publisher.
